# The Role of Advanced Imaging in Gout Management

**DOI:** 10.3389/fimmu.2021.811323

**Published:** 2022-01-14

**Authors:** Shuangshuang Li, Guanhua Xu, Junyu Liang, Liyan Wan, Heng Cao, Jin Lin

**Affiliations:** Department of Rheumatology, The First Affiliated Hospital, Zhejiang University School of Medicine, Hangzhou, China

**Keywords:** ultrasonography, DECT, MRI, gout, management

## Abstract

Gout is a common form of inflammatory arthritis where urate crystals deposit in joints and surrounding tissues. With the high prevalence of gout, the standardized and effective treatment of gout is very important, but the long-term treatment effect of gout is not satisfied because of the poor adherence in patients to the medicines. Recently, advanced imaging modalities, including ultrasonography (US), dual-energy computed tomography (DECT), and magnetic resonance imaging (MRI), attracted more and more attention for their role on gout as intuitive and non-invasive tools for early gout diagnosis and evaluation of therapeutic effect. This review summarized the role of US, DECT, and MRI in the management of gout from four perspectives: hyperuricemia, gout attacks, chronic gout, and gout complications described the scoring systems currently used to quantify disease severity and discussed the challenges and limitations of using these imaging tools to assess response to the gout treatment.

## Introduction

Gout is a chronic disease of monosodium urate (MSU) crystal deposition and is one of the most common forms of inflammatory arthritis in adults, especially men. The incidence and prevalence of gout are increasing worldwide, with recent data estimating that the prevalence of gout ranges from roughly <1% to 6.8% in Western countries and about 1.1% in China (1, 2). People with metabolic syndrome are more likely to have higher serum urate levels (3). Persistent serum urate levels (sUA, >360 μmol/l or >6 mg/dl) may lead to much MSU crystal deposition in tendons, joints, or other unusual tissues (4) and trigger acute joint inflammation (gout flares) or chronic joint inflammation (gouty arthritis and joint structural damage) (5). The typical symptoms of a gout flare are rapid painfulness, hotness, redness, and swelling in the joints (6), which are self-limiting inflammatory responses that usually disappear within 14 days. Alone or in combination, use of NSAIDs, colchicine, or corticosteroids is recommended in treating gout flares with an effective and rapid control of acute inflammatory attack. Urate-lowering therapy (ULT) is a long-term management of gout to reduce serum urate levels, which can lead to dissolution of MSU crystals deposition, reduction or prevention of gout attacks, and joint damage (7, 8). Although gout is “curable” through ULTs, this disease is poorly managed worldwide, because of poor adherence in gout patients to the medications (e.g., febuxostat or allopurinol) of ULTs.

MSU crystals from tophi or joint synovial fluid aspiration by microscopic detection are negatively birefringent and needle-shaped (9), which is still the gold standard in gout diagnosis, while with the rapid development of imaging techniques, particularly US, DECT, and MRI, which provide non-invasive and clear identification of MSU crystal deposition, they are considered to be a promising tool for gout diagnosis (10, 11). In 2015, the classification criteria recommended by the European League Against Rheumatism (EULAR) and the American College of Rheumatology (ACR) endorsed the US and DECT as a new and effective diagnostic tool for gout (12). What is more, these advanced imaging techniques can be used to assess the severity of gout and monitor the response to treatment in gouty patients. Although MRI is not specific enough in gout disease, it can be valuable in the assessment of soft tissue and bone damage in gout.

The main ultrasound imaging findings of gout include the double contour sign (DCs), tophus, aggregates, and erosion. These definitions were developed by the US group of Outcome Measures in Rheumatology (OMERACT) in 2015 (13). DCs are a deposit of urate on the surface of articular cartilage, forming two hyperechoic bands with the bone cortex, which are present regardless of the angle of the irradiation. Tophus refers to a large collection of hypoechoic or uneven hyperechoic MSU crystals. Aggregates are small inhomogeneous hyperechoic crystal depositions, and erosion is the discontinuity of the bone cortex and can be seen in two different vertical planes. DECT is a new imaging technique developed from conventional CT, which uses a dual-source scanner to irradiate two X-ray beams onto different materials for identification. Differences in the energy absorption curves of chemical entities are used to accurately calculate the composition of an object (14). The MSU deposits are often coded in green and can be seen in DECT images. MRI has good soft-tissue resolution and can show cartilage damage, soft tissue inflammation, and bone erosion very well. There are four main elements to the MRI assessment of gout, tophi, synovitis, bone marrow edema, and bone erosion (15). The rheumatoid arthritis magnetic resonance image scoring system (RAMRIS) is now commonly used to assess the disease progression of gout in MRI (16). This review mainly focuses on the role of US, DECT, and MRI in the management of gout.

## Hyperuricemia

Hyperuricemia is defined as a serum uric acid level above 7 mg/dl. Most people with asymptomatic hyperuricemia (AH) do not develop gout (17), and the predictors of the transition from hyperuricemia to gout are unknown, leading us to overlook the possibility that this population may benefit from early uric acid-lowering treatment. Recent studies have shown that US and DECT imaging techniques can be used to detect MSU deposits in asymptomatic hyperuricemia. The consistency between MSU crystals and US gouty features (DCs and hyperechoic areas) in asymptomatic hyperuricemia patients was first reported in 2012, validating the role of US in the early diagnosis and detection of structural damage in AH patients (18). Metatarsophalangeal 1 (MTP1) and femoral condyle were the most common US scan sites for DCs and tophi, and a high prevalence of gouty damage in AH patients was observed (19). MSU deposits could also be detected with DECT in AH patients, but they were larger and occurred significantly more frequently in gouty patients. This suggested that a certain threshold of MSU deposition may be required during the transition from AH to gout (20). 15% of AH individuals had subclinical MSU depositions on foot or ankles by DECT detection (21). These findings highlight the important role of subclinical MSU deposition in disease progression and the need to explore the clinical significance of crystals; the use of US and DECT may provide greater insight and understanding of asymptomatic hyperuricemia.

According to the current gout classification criterion put forward by EULAR and ACR in 2015, the pain characteristics of gout are acute onset, generally reaching the maximum pain within 24 h and lasting less than 14 days, which is an episode pain (12). However, clinically, we also encounter many symptomatic hyperuricemia patients who have persistent foot pain that does not fit typical gout. Little is known about this special population. Recently, a study included 16 patients with hyperuricemia and persistent foot pain as an experimental group, compared to 15 AH individuals (22). The experimental group was given 80 mg/day with febuxostat for 3 months; the US imaging and 24-h and 7-day visual analog scores were assessed in baseline, the first month (M1), and the third month (M3) after ULTs. The results showed that sUA and foot pain scores of patients in the experimental group decreased significantly under the treatment of febuxostat. Then, the experimental group was divided into two groups based on the presence or absence of DCs; further analysis demonstrated that DC-positive patients had obviously lower 24-h and 7-day pain scores at M3, but with no significant difference of sUA levels between the two groups. These results suggested that hyperuricemia patients who have sustaining foot pain and positive US features may be the alternative gouty presentation and responsive to the ULTs, which may also be included in the gout classification criteria to identify and treat gout at an earlier stage, increasing diagnostic sensitivity and treatment effectiveness.

## Gout Flares

Serum uric acid levels have long been considered the endpoint of conventional uric acid-lowering therapy. It was previously thought that the altered sUA concentrations were a risk factor of flares (23), and controlling serum uric acid levels could prevent gout attacks. However, this has been challenged by several studies, including those from ACR, who have argued that the correlation between targeting sUA and gout flares reduction was inadequate (24–27). In the febuxostat trial, for example, there was no significant reduction in gout attacks compared with placebo, even when the uric acid level was controlled (28, 29). Similarly, in a 6-month randomized controlled ULT study, the frequency of gout attacks actually increased (30). Thus, there is a missing part between sUA and gout flares, and recent studies suggested that MSU crystals might be the key link between them. It has been hypothesized that the decrease in serum uric acid leads to the instability and dissolution of urate, exposing it to the autoimmune system and producing a strong immune response that leads to an outbreak of gout (23). Therefore, the volume and level of urate crystals are closely related to the onset of gout and need to be monitored continuously, which is also a good way to guide treatment to reduce uric acid.

In a follow-up observational study of 62 patients under ULTs, their MSU deposits were assessed by DECT and US, suggesting that MSU crystal burden may be a predictive risk of gout flares (31). Patients attended the visits at 0, 3, 6, and 12 months, and their knees and feet were all scanned. The study revealed that the presence and volume of urate deposits in feet measured by DECT were significantly associated with flares, rather than the number of joints bearing DCs assessed by US. The analysis of DECT evaluation displayed that for every 1-cm^3^ increase in urate deposition volume in feet, the risk of gout attacks increased 2.03-fold during the first 6 months compared to the baseline. Moreover, the optimal threshold for differentiating the patients with or without gout flares was 0.81 cm^3^ in this research. All these data verified the concept that urate burden was related to the risk of gout flares. Interestingly, the change in sUA levels from M0 to M6 was not obviously different among the participants undergoing or not undergoing gout flares. The role of DECT in the management of gout was supported, beyond diagnosis. It may be decisive to assess MSU burden by using DECT to identify which one was still at a high risk of gout flares when considering maintain or interruption of ULTs, especially for patients who have reached the targeted sUA level but still have urate deposition.

In the treatment of gout, a basic principle is to prevent the onset of gout in the initial stage of ULTs, but there is no consensus on how long prophylaxis drugs should be used to prevent gout attacks during ULTs. EULAR recommends at least 6 months of prophylaxis (e.g., NSAIDs or colchicine) (7), while ACR recommends that 3–6 months of prophylaxis should be followed, and screening for gout activity and continued use of anti-inflammatory drugs are needed if patients have a recurrence of gout after cessation (8). A recent study including 79 patients might shed some light on this question (32). The study was divided into two stages: the first stage was a 6-month ULTs with gout attack prophylaxis, and the second phase was 6 months of ULT maintenance therapy with stopped gout prophylaxis. Nearly half of the patients in this study had at least one episode of gout within 6 months of discontinuing their gout prophylaxis drugs; the high incidence was in line with existing research (28, 29, 33). The authors found that DCs, tophi, and sUA levels all changed significantly after treatment. Among them, DCs were the earliest indicator that changed (appear at M3). However, changes in sUA were not associated with the prevalence of gout attacks, which was consistent with other results that there was no confirmed relationship between sUA and gout flares (30, 34). Interestingly, the low rate of gout relapse was found in patients with a greater than 50% reduction in tophi volume, suggesting that changes in tophi volume could be a predictor of gout onset. These results might provide some ideas for research on the duration of gout prophylaxis and emphasize the big role of MSU crystal depositions in gout flares. It is reasonable that follow-up of MSU depositions with US and DECT can help physicians predict the onset of gout and discontinue gout prophylaxis at an appropriate time.

In addition to the four main features of US in gout mentioned above, the altered Doppler flow signal, which is unique to US, can also clearly show the inflammatory signs of an acute attack of gout. The ultrasound Doppler flow signal is more suggestive of an acute attack of gout than an assessment of the clinical presentation of the gout patient (35). It has been suggested that the ultrasound Doppler signal is more pronounced during an acute gout attack than during the intercritical phase (36). In addition, Doppler ultrasound can also indicate the responsiveness of gout patients to ULT. A study revealed that after more than 2 years of ULT, the Doppler signal was still present in a large number of gout patients, although it has been reduced to varying degrees. This finding raised reflections on the accuracy of current outcome measures and treatments (37). These data show that ultrasound Doppler signaling also played a role in the assessment of gout progression.

## Chronic Gout

### DECT

There is growing interest in the important role of MSU deposition in the development of gout. Many studies have concluded that MSU crystals were the key pathology of gout. Instead of merely controlling serum uric acid levels, treatments that address urate crystals are truly effective. Importantly, the link between sUA and MSU crystals was weak (38), which made it difficult to assess the dynamics of MSU depositions by just measuring sUA levels. Such a view manifested the importance of monitoring MSU deposits during ULTs, and DECT was an appropriate approach. DECT has a high sensitivity in detecting even very small urate crystals and allows the testing of some special sites such as tenders, spine, blood vessels, which cannot be measured by other imaging or joint fluid aspiration (11). Furthermore, DECT allows automatic calculation of uric acid crystal volume for quantitative analysis, which is an important parameter in the outcome of the disease and can better evaluate the treatment from the starting point to the follow-up (39).

Allopurinol is the first-line drug for the treatment of gout, and DECT can visualize the therapeutic effect of allopurinol on serum uric acid levels and MSU depositions. A prospective study recruited 152 patients treated with allopurinol ≥ 300 mg/day for at least 3 months and then used DECT to assess the total number and volume of crystal deposition (40). The result showed that uric acid deposition occurred in approximately 69.1% of patients despite a mean of 5.1 years of treatment with allopurinol 300 mg/day or higher. More specifically, the presence of MSU deposition was 90% among patients with sUA ≥6.0 mg/dl and tophus and 46.9% among those with sUA <6.0 mg/dl and without obvious tophi. DECT demonstrated that the volume of the MSU crystal was greater in those with sUA ≥6.0 mg/dl, and higher crystal deposition was positively related to higher gout flares, more tophi, and worse disease activity scores in patients. In this paper, DECT detection confirmed that nearly half of the patients still had urate deposition even though the serum uric acid level had been controlled (<6.0 mg/dl), prompting us to consider whether more intensive ULTs was needed for better gout control.

Another longitudinal study of 77 patients was undertaken to assess MSU deposition depletion by DECT under the treatment of conventional ULT drugs or lifestyle (41). The first choice was allopurinol with the dose of 100 mg/day initially and gradually increased to the maximum dose of 600 mg/day if sUA was not satisfied. Participants who were intolerable to allopurinol were treated with the drug febuxostat in 80 mg/day and gradually titrated up to 120 mg/day to control the sUA level. The result showed that both the lifestyle and ULTs were useful in dissolving MSU deposits after 18 months. Urate precipitation dissipated the most in the febuxostat group, followed by the allopurinol group, and finally in the lifestyle improvement group, suggesting the better effect of febuxostat than allopurinol in MSU depletion. Moreover, it was encouraging that these data confirmed the use of lifestyle intervention on MSU decline, beyond preventing gout flares. In a word, it is necessary to observe whether urate crystals are completely relieved through DECT under long-term treatment of ULTs.

Recurrent gout attacks and chronic gout inflammation can lead to severe structural damage to the painful bones, causing great harm to patients. How to reduce bone damage in the treatment of gout is a very important and urgent issue to be addressed. A randomized controlled trial of 87 patients (dose-escalation group, n = 42, control group, n = 45) discovered a benefit of allopurinol escalation on bone damage by DECT detection (42). Patients in the dose-escalation group were initially treated with increased allopurinol to rapidly reach the targeted sUA. On the other hand, the controlled group was treated with a conventional dose of allopurinol in Year 1, followed by an increased dose of allopurinol in Year 2. The DECT data suggested that the strategy of allopurinol dose escalation prevented bone erosion compared with the control group after a 2-year treatment, although the change was small. However, the result of XR imaging did not show any differences between the two groups. These findings confirmed that gout treatment was a long-term battle. The study of structural damage in gout joints is a huge challenge, and we need advanced imaging methods such as DECT to monitor the treatment effectiveness with its better sensitivity than plain radiographs. Also, the reduction in the volume of MSU crystals was much larger compared to the small change in bone erosion, revealing a big lag between the deposition disappearance and radiographic changes. This study suggested that high levels of allopurinol might facilitate bone reconstruction in gouty joints, and other cytological studies support this concept. Allopurinol and oxypurinol drugs could promote the differentiation and development of osteoblasts and promote bone repair (43). The mean sUA level here was 0.33 mmol/l, and the results showed slight prevention of bone destruction. In contrast to the result, another research showed that serum uric acid level was undetectable under pegloticase treatment and a significant improvement in bone erosion was observed (44). Taken together, lower serum uric acid levels may be required to reverse or improve bone erosion.

Pegloticase is recommended for patients with severe intractable gout, which could cause a dramatic drop in sUA level (45). A previous study was conducted using pegloticase in 10 patients by DECT. The data demonstrated that both sUA levels and tophi were very sensitive to pegloticase, with 71.4% of tophi disappearing after a few weeks, especially in the joints, while tendon tophi were broken down more slowly (45). Similar results were found in another paper. In a patient with refractory gout, 6 months of treatment of pegloticase resulted in a significant reduction in tophi. At the same time, compared with physical measurement and US detection, DECT reflected changes in urate volume better (46). To sum up, DECT is a remarkable imaging means for a comprehensive assessment of MSU burden and longitudinal monitoring of the response to ULTs. Moreover, the state of being tophi-free can be measurable and perceptible by DECT, even for those refractory gouty patients when the right medicine is used.

In addition to ULTs, anti-inflammatory treatment should be administered at the same time, which is very beneficial for gout management. Both soft tissues and urate crystals in tophi are closely related to bone damage. Cytological studies have found that soft tissues surrounding tophi mainly contain immune cells, chemokines, cytokines, and osteoclasts, forming such a chronic inflammatory microenvironment (47, 48). Among them, mononuclear macrophages play a major role in promoting the growth and development of osteoclasts by secreting COX-2, PGE2, IL-1β, and TNF (49). Meanwhile, osteoblasts, which play a role in bone remodeling and bone repair, are significantly inhibited in their activity, function, and morphology by MSU crystals (50). A study led by conventional CT, DECT, and XR techniques showed that urate crystals and soft tissue composition of tophi were directly associated with bone destruction scores, a significant reduction in soft tissue inflammation was associated with improved bone destruction after ULTs (51). These data further intuitively confirmed the role of inflammatory soft tissues in the progression of gout disease, reflecting the value of ULTs in combination with anti-inflammatory drugs or the latest biological agent intervention. Moreover, imaging is of great value in monitoring these therapeutic effects.

### US

The MSU load was identified by the OMERACT working group as one of the therapeutic targets in gout management (52), and the reduction in urate deposition can also be demonstrated by US imaging, which mainly includes four features of gout lesions. In a related study, 79 patients were treated with ULTs and underwent US monitoring of MSU deposition over a 6-month period (53). The results showed that DCs and tophus features on US were significantly reduced during treatment. Among them, the DCs could be an early marker with an obvious change after 3 months of treatment, while tophi changed more significantly after 6 months of treatment. In line with other studies (54, 55), the reduction in urate deposition was associated with low sUA levels and was more pronounced in the group with lower serum sUA (<5 mg/dl), suggesting that lower levels of sUA were one of the effective therapeutic targets and consistent with last EULAR recommendations (7). Similarly, several other research also showed that the DCs, tophi, and aggregates decreased obviously measured by US under ULTs (55–57).

The concept of treat-to-target (T2T) is well established in many chronic diseases, including rheumatism, and the first T2T recommendations for gout were put forward by EULAR in 2016, ushering in a new era of targeted treatment for gout (7). One of the first principles is to keep the patient’s serum levels below the target level throughout the whole life to eliminate urate crystal deposition. This strategy also provided some remission criteria for gout patients, but there were some limitations. For example, the proposals do not provide substantive recommendations on clinical practical issues such as the optimal target value of blood uric acid and imaging criteria for remission.

In 2020, one of the largest US studies revealed the imaging changes with a T2T approach. The study manifested an apparent crystal dissolution in 209 gout patients by adopting a T2T way in ULTs (58). The DCs were found to be the most sensitive and earliest variable marker, which was consistent with the results of other paper (53). At 12 months, nearly half of the patients had no DCs here. A reasonable explanation for the early disappearance of DCs may be that MSU deposits are in close contact with cartilage and joint fluid. During the progress of ULTs, serum uric acid levels fall rapidly, causing the reduction of the uric acid levels in joint fluid to equilibrate them with blood concentrations. They also found that MTP1 joints were the most common site of MSU depositions, and the erosion in MTP1 was highly related to the three other sonographic findings. Meanwhile, US results indicated that about 1/6 of the participants had DCs on proximal talar cartilage and distal femur and had tophi in distal patellar tendon and triceps, showing that these sites also were involved in the development of gout. This US study provided an important result that gouty patients who followed a T2T approach in ULTs in clinical practice significantly reduced the MSU crystal burden.

Another study also used OMERACT defined US gout lesions to assess MSU changes after ULTs. The study examined a number of sites in each patient, including 28 joints and 26 tendons, to better understand urate depositions (59). The results showed that MTP1 was the most prone to MSU deposition, followed by MTP2-4s and the knee joint. Importantly, they found that the most easily affected by MSU crystals were also the site of the best treatment response. Detection of so many locations by US was time-consuming, but it was meaningful to determine which sites were significant in gout diagnosis and treatment monitoring. Here, the mutual involvement and rapidly effective responsiveness to ULTs in MTP1–3s and knee joints display the possible essential of choosing these positions. Consistent with the results mentioned above, the authors also found that the changes of DCs were most sensitive, with a significant reduction even in the first 3 months of treatment. More interestingly, the study also observed obvious improvement in inflammation response through US imaging and biological markers. The results indicated that the synovitis and tenosynovitis were significantly improved; the inflammatory molecule CRP was dramatically decreased.

To date, there were still no clear criteria for which locations and at least how many areas are needed for more accurate gout diagnosis and treatment monitoring. Different groups chose different sites for imaging measures of MSU deposits. One of the groups identified the bilateral evaluation of the radiocarpal joint, two tendons (patellar tendon and triceps tendon) for aggregates, and three articular cartilages (including MTP1, second metacarpal, and talar) for DCs, having a satisfying sensitivity and specificity in gout diagnosis (60). Another group reported that the scanning of two knees and two MTP1 for DCs and aggregates was sufficient (61).

### MRI

A 2-year study enrolling 314 individuals revealed the protective effects of febuxostat on the joints of patients with early gout (62). This was the first randomized controlled trial of ULTs in early gout patients to use imaging to assess the damage within the gouty joints. At baseline, there was little evidence of joint erosion, and after 2 years of observation, there were no significant changes in joint erosion or joint space narrowing in either the experimental or the control group of patients. This suggested that damage to joint structures may be a manifestation of advanced gout. Importantly, the presence of synovitis was initially detected by MRI in most subjects, and after 2 years, patients in the febuxostat group had significantly better RAMRI synovitis scores and significantly fewer acute gout attacks compared to the placebo group. The clinical significance of synovitis in gouty patients is unclear, and whether synovitis is a risk factor for gout flares or joint damage has yet to be investigated. The results of this study showed that febuxostat improved intra-articular synovitis and also reduced the frequency of acute gout flares, providing some indication of the role of synovitis in disease progression. Febuxostat not only reduced serum uric acid levels but also had a better therapeutic effect on early gouty synovitis, suggesting the need for early treatment of gout.

Uric acid deposits in the knee joint lead to restricted knee movement and can seriously affect the patient’s daily life. A study has shown that ULTs can significantly reduce gout tophi deposits in the knee joint (63). The study used MRI to assess the size of gout tophi within the patient’s knee joint and vernier calipers to measure the size of subcutaneous gout tophi throughout the body and continued to assess intra-articular and subcutaneous gout nodules under 18 months of ULTs. The results showed a significant improvement in knee mobility after ULTs, which also correlated positively with a reduction in intra-articular tophi. Further analysis revealed that increased knee mobility was also significantly associated with a reduction in subcutaneous tophi. Combining these data, and taking into account the relatively expensive nature of MRI, we were able to use subcutaneous tophus nodules to assess the dissipation of gout tophi within the knee joint in order to predict and monitor improvements in knee mobility. Knee gaps were still present within the knee joint in the patients included in this study, suggesting that aggressive application with ULTs prior to irreversible knee destruction could significantly improve knee motion and avoid permanent joint damage.

In conclusion, imaging methods are well managed and monitored at all stages of gout disease progression (**Table 1**).

**Table 1:** The role of imaging in monitoring treatment in the different stages of gout.

		Management or treatment of gout					
Stages	Imaging modality	Experimental group	Control group	Outcome	Scanned sites	Follow-up visit	Ref.
Hyperuricemia	US	16 patients (hyperuricemia and persistent foot pain) with 80 mg/day febuxostat	15 individuals with AH	Sustaining foot pain and DCs positive patients had obviously lower pain scores under ULTs	MTP1	M1, M3	(21)
Gout flares	DECT	62 gouty patients under allopurinol/febuxostat	–	Every 1-cm^3^ increase in MSU volume in feet, the risk of gout attacks increased 2.03-fold	Feet	M3, M6, M12	(30)
	US	79 individuals with a 6-month ULT and gout prophylaxis	79 individuals with continuous 6-month ULTs and stopped prophylaxis	The low rate of gout relapse was found in patients with a greater than 50% reduction in tophi volume, s	MTP1, knees	M6, M12	(31)
Chronic gout	DECT	152 patients with allopurinol ≥ 300 mg/day for a mean of 5.1 years	–	The volume of MSU crystal was greater in those with sUA ≥ 6.0 mg/dl and tophi	Hands/wrists feet/knees	Day 1 Day 28	(36)
	DECT	77 gouty patients with lifestyle improvement or allopurinol or febuxostat	–	Urate precipitation dissipated the most in the febuxostat group, followed by allopurinol group, and finally in the lifestyle improvement group	MTP1, toes Feet/ankle Soft tissues	M18	(37)
	DECT	42 patients with dose escalation of allopurinol during 2 years	45 patients with no change dose of allopurinol at Year 1 and dose escalation in Year 2	Higher levels of allopurinol benefits bone reconstruction in gout joints	Feet	Year 1, Year 2	(38)
	DECT	10 refractory gouty patients under 8 mg/day pegloticase intravenously every 2 weeks	–	sUA levels and tophi were both sensitive to pegloticase, 71.4% of tophi disappeared	Hands/wrists feet/ankles	Mean of 13.3 weeks	(41)
	DECT	A patient with refractory gout with a 6-month pegloticase	–	A significant reduction in tophi	Hands and feet	M6	(42)
	US	79 gouty patients under allopurinol/febuxostat	–	DCs and tophi features were significantly reduced	Knees and MTP1s	M3, M6	(49)
	US	209 gouty patients under allopurinol/febuxostat	–	The T2T under ULTs can reduce all MSU depositions, especially for DC	Hands/wrists feet/knees	M3, M6, M12	(54)
	US	50 gouty patients with allopurinol/benzbromarone/febuxostat	–	A significant deduction of DC, tophus and aggregate sum scores	28 joints and 26 tendons	M3, M6	(55)
	MRI	157 early stage gouty patients with febuxostat	157 early-stage gouty patients with placebo	Patients on febuxostat had better RAMRI synovitis scores and significantly fewer acute gout attacks	Hands and feet	M6, M12 M18, M24	(58)
	MRI	26 patients with tophaceous gout and limited knee motion	–	A significant improvement in knee mobility and a reduction in intra-articular tophi	Knees and all subcutaneous nodules	M18	(59)

## Gout Complications

Severe gout is often accompanied by various complications, such as diabetes, obesity, cardiovascular disease, and kidney disease, especially in terms of damage to the heart and kidney organs (2).

Currently, there were many studies on the relationship between gout and coronary heart disease. It has been suggested that gout can significantly increase the incidence of coronary heart disease (64). In acute coronary syndromes, high sUA levels have been found to significantly increase the risk of coronary heart disease (65). Moreover, there were also some contradictory conclusions: one study showed no significant association between gout and myocardial infarction (66), and another suggested that urate deposition burden on knees and feet in gouty patients did not increase the risk of cardiovascular events (67). However, none of these directly indicated the cardiovascular system with gout involvement. A recent study of 59 gouty patients and 47 controls visually illustrated the MSU deposits on the coronary artery by DECT. The results showed that patients with gout had more MSU deposits on coronary arteries and were also found to have more coronary calcification (68). Urate deposits in this study were confirmed by polarizing microscopy, rather than the so-called artifacts seen in other studies.

For the renal manifestations of gout, both US and DECT can detect MSU deposition in the renal medulla of patients with severe gout (69). The former one was characterized by strong echogenicity in the renal medulla accompanied by a posterior acoustic shadow, while the latter could analyze the composition of deposits. US is commonly used to detect urolithiasis in gout, but also its usefulness in gouty nephropathy, especially its cheapness and ease to be used compared to DECT.

A large cross-sectional study also confirmed the role of US in detecting the changes in the kidney induced by gout. They found that 36% of 502 untreated gouty patients showed hyperechoic uric acid crystal deposits in the renal medulla by US, while none was observed in the 515 controls (70). Multivariate analysis found that hyperechoic patterns in the medulla were associated with gout arthritis, DC thickness, disease duration, and reduced glomerular filtration rate. These findings provided a basis for gout-induced microcrystalline nephropathy and also provided a new therapeutic target for gout.

**Figure 1:**
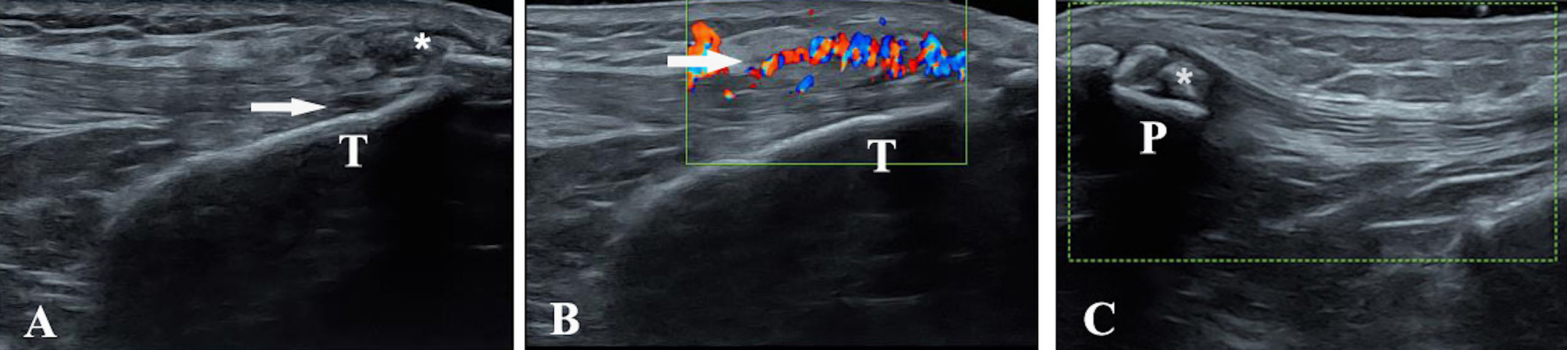
Ultrasound evaluation in gouty patients. Longitudinal scans of the patellar ligament and the quadriceps tendon in B-mode and power Doppler ultrasound. **(A)** The asterisk shows the tophus of the patella ligament. **(B)** Doppler flow signals indicate acute inflammation of quadriceps tendon. **(C)** The asterisk shows the tophus of the quadriceps tendon.

In addition, MSU can be deposited in some unusual areas such as the ribs, spine, wrists, and lower extremity enthesopathy, causing pain to the patient. Many recent reports in the literature have found evidence of gout involvement in the spine of patients by means of DECT and MRI, as a means of identifying the cause of their pain and also allowing assessment of the efficacy of ULTs on MSU deposits in the spine and reducing unnecessary surgery (71–73). It was noted that MRI showed that some gouty patients already had significant synovitis and bone erosion at the time of the first acute gout attack, suggesting that damage to the involved joints had already occurred during the subclinical period of gout (74). Our previous study showed that the lower-extremity enthesopathy was also a location that could accumulate in gouty patients but was often overlooked, and we found that the patellar ligament, quadriceps femoris tendon, and quadriceps patellar insertion site were very likely to be deposited by MSU. Moreover, Doppler flow signals indicated acute inflammation of the quadriceps tendon (75) (**Figure 1**). The management and monitoring of gout complications are an important part of gout treatment; by using modern and advanced imaging methods, we can clearly know MSU depositions so as to better understand disease development and dynamic manifestations.

## Imaging Limitations and Challenges

Overall, imaging methods play an important role in the treatment and management of gout, but there are some limitations. Artifact is common in DECT, especially in nail beds, nose, skin, and peroneal tendons, leading to false-positive results (76, 77). A study displayed that compared to the parameter of 130 HU, adding a tin filter and setting a minimum attenuation of 150 HU could effectively reduce artifacts (78). In addition, DECT cannot effectively evaluate the internal structure of the joint including cartilage, ligament, tendon, and synovial membrane. Compared to US, DECT and MRI are relatively expensive. Moreover, the machine is not easy to move, which has limitations for patients with acute attacks. US is cheaper and more convenient than DECT or MRI. However, unlike DECT, urate deposition volume on US cannot be calculated automatically. The evaluation of crystal volume by US is related to the experience and ability of the readers. In addition, there is a large heterogeneity in the ultrasound instruments and probes used, which might affect the consistency and accuracy of image results. When we use DECT and US imaging methods to monitor the dispersion of crystal deposition under ULTs, we must take into account that DECT only shows the MSU crystal itself, whereas US fully shows the entire tophus volume, including the inflammatory tissues around the crystals, as confirmed by some histological analysis (39, 79). Therefore, the evaluation of DECT for tophi is usually smaller than US. At the same time, we should also note that the tophi often attach to the surface of the joint and the measurement of depth by US is affected by acoustic shadow, leading to the overestimation of its volume by US. On the other hand, compared to US, MRI has better visualization of deep structures and can image multiple planes for a single part, which shows disease pathology in depth.

## Imaging Scoring Systems

These advanced imaging have a certain role in evaluating the effect of ULTs, but the current studies are scattered and independent, making it difficult to combine all imaging findings. We need a suitable scoring system with good reproducibility and sensitivity to obtain standardized results. Currently, available US studies use either a binary scoring system or a semiquantitative scoring system. A binary scoring system means that a patient is defined as 1 if they have one of the US features (DCs, tophus, aggregates, or bone erosion) and 0 if they do not (59). A semiquantitative score is defined in terms of the degree to which the US features are present: 0 = none, 1 = a little, 2 = sure, and 3 = large (58). Both scoring systems were found to be sensitive to changes during treatment. However, it should be noted that both scoring methods may overlook minor lesions during follow-up.

Two related studies referred to a scoring system for DECT imaging (41, 80) and proved to be sensitive and effective. The DECT scoring system mainly consists of 4 regions: 1) MTP1 joints; 2) toes; 3) midfoot and ankle joints; and 4) soft tissues (tendons). Results were scored based on the maximum amount of MSU depositions at each site, each scored from 0 to 3 (0 = none, 1 = little dots, 2 = deposit >2 mm, 3 = fused depositions). Moreover, the ultimate score was obtained by adding the scores of the four areas, with a maximum score of 12. The inter-reader correlation coefficient (95% confidence interval) for the DECT MSU deposit score was 0.98 (0.97–0.98). This method could be used to detect the most affected areas of the crystal, and the results were highly consistent with the full-scan results and required significantly less time. Not only is it effective in distinguishing people with and without gout, but also it clearly identifies whether they are responsive to ULTs.

More research is needed to validate the above method, and in the meantime, future research should try to explore whether a more sensitive and accurate scoring system can be found.

## Conclusions

Advanced imaging technologies allow us to visualize the pathological process of tissue destruction in gout, providing a new way to explore the disease *in vivo*. The use of US, DECT, and MRI is beneficial for earlier gout diagnosis and monitor of patients’ response to ULTs and anti-inflammatory drug treatment. The combination of clinical manifestations, laboratory indicators, and imaging techniques can improve the understanding and adherence to treatments of gouty patients, improve their prognosis, reduce complications, and improve their quality of life. At the same time, we need to consider the challenges and limitations of using these imaging technologies. We need a better scoring system and the ultimate location of image examination to explore the optimal serum urate acid levels and the best monitoring methods for gout management.

## Author Contributions

SL and HC conceived of the study and its design and drafted the manuscript. GX and LW provided the gouty pictures. JYL, HC, and JL participated in its coordination and modification. All authors contributed to the article and approved the submitted version.

## Conflict of Interest

The authors declare that the research was conducted in the absence of any commercial or financial relationships that could be construed as a potential conflict of interest.

## Publisher’s Note

All claims expressed in this article are solely those of the authors and do not necessarily represent those of their affiliated organizations, or those of the publisher, the editors and the reviewers. Any product that may be evaluated in this article, or claim that may be made by its manufacturer, is not guaranteed or endorsed by the publisher.

## Abbreviations

ACR, American College of Rheumatology; AH, asymptomatic hyperuricemia; DCs, double contour sign; DECT, dual-energy computed tomography; EULAR, European League Against Rheumatism; M1, first month; M3, third month; MRI, magnetic resonance imaging; MSU, monosodium urate; MTP1, metatarsophalangeal 1; OMERACT, Outcome Measures in Rheumatology; RAMRIS, rheumatoid arthritis magnetic resonance image scoring system; ULT, urate-lowering therapy; US, ultrasonography.
